# Supplementary material for: Effects of low pH and feeding on calcification rates of the cold-water coral Desmophyllum dianthus
Source: PeerJ. 2020 Jan 2;8:e8236. doi: 10.7717/peerj.8236 (PMC6942680; doi:10.7717/peerj.8236)
Supplement: Supplemental Information 2 [file peerj-08-8236-s002.pdf]

Supplementary material

Table S1. Estimates of abundance and concentration of food particles expressed as particles L^{-1} (*Cyclops, Artemia* and *Mysis*) in 10L aquaria. Concentrated food was previously resuspended in 40mL of seawater. Sample size = 3mL, N = 6.

	Abundance	SD	SE	Density
Cyclops	915	79	32	91.5
Artemia	35	13	5	3.5
Mysis	17	5	2	1.7
